# High glucose activates the alternative ACE2/Ang-(1-7)/Mas and APN/Ang IV/IRAP RAS axes in pancreatic β-cells

**DOI:** 10.3892/ijmm.2013.1469

**Published:** 2013-08-14

**Authors:** CARMEN HÄRDTNER, CAROLINE MÖRKE, REINHARD WALTHER, CARMEN WOLKE, UWE LENDECKEL

**Affiliations:** Department of Medical Biochemistry and Molecular Biology, University of Greifswald, D-17475 Greifswald, Germany

**Keywords:** renin-angiotensin system, alternative renin-angiotensin system, β-cell, high glucose, BRIN-BD11 cells

## Abstract

The activation of the classical angiotensin (Ang)-converting enzyme (ACE)/Ang II/Ang II type 1 receptor (AT1R) axis of the renin-angiotensin system (RAS) has been associated with islet dysfunction and insulin resistance. Hyperglycaemia, hypertension and obesity, major components of metabolic syndrome, are all associated with increased systemic and tissue levels of Ang II. Whereas it is well established that Ang II, by binding to AT1R, impairs glucose-stimulated insulin secretion and insulin signaling, the contribution of alternative RAS axes to β-cell function remains to be fully elucidated. In this study, using the BRIN-BD11 rat insulinoma cell line, we i) examined the basal expression levels of components of classical and alternative RAS axes and ii) investigated the effects of normal (5.5 mM) and elevated (11, 15, 25 mM) glucose concentrations on their expression and/or enzymatic activity by means of reverse transcription quantitative PCR (RT-qPCR), immunoblot analysis and enzymatic activity assays. The results correlated with the insulin production and release. Essential components of all RAS axes were found to be expressed in the BRIN-BD11 cells. Components of the alternative RAS axes, ACE2, neutral endopeptidase 24.11, Mas receptor (Mas), aminopeptidases A (APA) and N (APN) and insulin-regulated aminopeptidase (IRAP) showed an increased expression/activity in response to high glucose. These alterations were paralleled by the glucose-dependent increase in insulin production and release. By contrast, components of the classical RAS axis, ACE, AT1R and Ang II type 2 receptor (AT2R), remained largely unaffected under these conditions. Glucose induced the activation of the alternative ACE2/Ang-( 1 - 7 )/Mas and APN/Ang IV/IRAP RAS axes simultaneously with the stimulation of insulin production/release. Our data suggest the existence of a functional link between the local RAS axis and pancreatic β-cell function; however, further studies are required to confirm this hypothesis.

## Introduction

Diabetes mellitus (DM) is a ~3,000-year-old disease, already dating back to the Egyptian era ( 1 ) . In 2011 ~366 million individuals worldwide suffered from diabetes and an estimated number of ~553 million persons will be affected by 2030 ( 2 ) .

Since 1936, DM is classified into insulin-dependent DM (IDDM) [nowadays known as type 1 DM (T1DM)] and non-insulin-dependent DM (NIDDM) [currently referred to as type 2 DM (T2DM)] ( 3 ) . The latter form, which is characterised by a relative lack of insulin, is much more common comprising ~90–95% of DM cases, whereas T1DM comprises the residual ~5% of all DM cases ( 4 ) . The relative insulin deficiency in T2DM is mainly due to the insulin resistance of target tissues, secretory defects and/or failure of the receptors ( 5 ) . A combination of genetic predisposition and lifestyle contribute to the prevalence of T2DM ( 6 ) . Common risk factors for T2DM include physical inactivity, smoking, alcohol abuse and environmental toxins, such as bisphenol A. In addition, hypertension, obesity and hyperglycaemia, 3 major hallmarks of metabolic syndrome, have been associated with increased plasma levels of angiotensin(Ang) II, a central component of the renin-angiotensin system (RAS) ( 7 – 9 ) .

**Figure 1:**
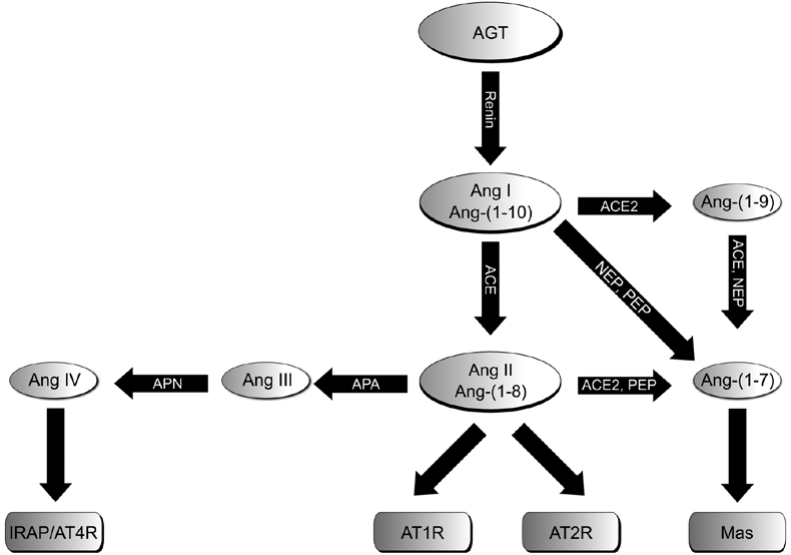
Schematic illustration of RAS consisting of the classical Ang II/ACE/AT1R axis and AT2R, as well as the two alternative axes, ACE2/Ang-( 1 - 7 )/Mas and APN/Ang IV/IRAP. RAS, renin angiotensin system; AGT, angiotensinogen; Ang, angiotensin; AT1bR, angiotensin II type 1b receptor; AT2R, angiotensin II type 2 receptor; ACE, angiotensin-converting enzyme; NEP, neutral endopeptidase 24.11; PEP, prolyl endopeptidase; Mas, Mas receptor; APA/APN, aminopeptidases A and N; IRAP, insulin-regulated aminopeptidase.

RAS, in particular Ang II, has been implicated in the onset and progression of T2DM. Systemic RAS is an endocrine system mainly known for its role in the regulation of blood pressure, fluid and electrolyte balance, as well as in volume homeostasis through different active metabolites. From the precursor of these active peptides, angiotensinogen, a decapeptide (Ang I) is cleaved off by renin ( 10 ) . A key enzyme of RAS, angiotensin-converting enzyme (ACE) then converts Ang I into the biological active octapeptide, Ang II, which preferentially binds to its receptors, Ang II type 1 receptor (AT1R) or Ang II type 2 receptor (AT2R) ( 11 ) . AT1R-mediated signaling leads to vasoconstriction, increased production of aldosterone and the secretion of vasopressin and in addition, promotes responses, such as inflammation, proliferation, fibrosis and atherosclerosis. On the contrary, AT2R signaling often exerts opposite effects, such as vasodilation or growth inhibition ( 12 , 13 ) . With the discovery of the ACE homologue, ACE2 in the year 2000, and the subsequent recognition of its crucial role in the generation of the Ang peptide, Ang- ( 1 - 7 ) , an important alternative RAS axis has been established ( 14 , 15 ) . The effects of Ang- ( 1 - 7 ) , which can also be produced by prolyl endopeptidase (PEP) or neutral endopeptidase 24.11 (NEP), are mediated by its putative receptor, Mas ( 16 , 17 ) . These effects are anti-proliferative, anti-fibrotic, anti-thrombotic and anti-arrhythmogenic; therefore, they generally oppose the effects of the classical ACE/Ang II/AT1R axis ( 18 ) . A second alternative RAS axis consists of the aminopeptidases A (APA) and N (APN), which successively convert Ang II into Ang III and then into Ang IV. In addition to its function in Ang peptide processing, APN has been implicated in the regulation of immune cell function ( 19 – 21 ) . Ang IV binds to the Ang IV receptor (AT4R) that has been identified as insulin-regulated aminopeptidase (IRAP). Similar to APN, IRAP also serves multiple functions in different organs. In adipocytes and muscle, IRAP is co-localised with the insulin-responsive glucose transporter, GLUT4, and is redistributed from the endosomes by GLUT4 specialised vesicles (GSVs) to the cell surface in response to insulin ( 22 , 23 ) . This translocation of IRAP to the cell membrane has been shown to be impaired in patients with T2DM ( 24 ) . Further evidence linking the APN/Ang IV/IRAP axis to glucose homeostasis includes decreased basal and insulin-stimulated glucose uptake into muscle and fat in IRAP-deficient mice ( 25 ) . Furthermore, Ang IV or its more stable analogue, Nle-Ang IV, have been reported to stimulate insulin secretion in INS-1 cells, and to reduce the increase in blood glucose during a glucose tolerance test or to improve insulin signaling in diet-induced hyperglycaemic mice ( 26 , 27 ) . The different signaling pathways of RAS are illustrated in Fig. 1 .

Local tissue-specific RAS acts independently from circulatory RAS, but can interact with the latter in an endocrine manner ( 28 , 29 ) . This has been shown to exist in the pancreas, including the islets of Langerhans ( 30 – 32 ) . A close association between RAS activation and diabetes has been confirmed by clinical trials showing the beneficial effects of ACE inhibitors (ACE _i_ s) and AT1R blockers (ARBs) on the incidence of DM, as well as on the reduction of cardiovascular complications in patients with DM [Heart Outcomes Prevention Evaluation (HOPE) and ONTARGET studies among others] ( 33 , 34 ) . Mechanistically, Ang II, by AT1R, impairs the phosphorylation of insulin receptor substrate 1 (IRS-1) by alternative phosphorylation on a serine, instead of a tyrosine residue, thereby decreasing phosphatidylinositol 3-kinase (PI3K) activity and enhancing mitogen-activated protein kinase (MAPK) pathways ( 35 ) . This diminishes insulin secretion and enforces insulin resistance. Accordingly, these detrimental effects of Ang II can be abolished by ARBs and/or ACE _i_ s ( 35 , 36 ) . Similar beneficial effects on insulin signaling and hyperglycaemia resulting from the blockade of the ACE/Ang II/AT1R axis have been demonstrated for Ang- ( 1 - 7 ) and the concomitant activation of the alternative ACE2/Ang- ( 1 - 7 ) /Mas axis ( 37 ) . This view is supported by studies demonstrating that Ang- ( 1 - 7 ) prevents metabolic syndrome and improves insulin resistance ( 38 , 39 ) . In adipocytes, Ang- ( 1 - 7 ) /Mas has been shown to affect glucose uptake and to suppress the production of reactive oxygen species (ROS) ( 40 ) .

In this study, using the BRIN-BD11 rat insulinoma cell line, we examined the expression/activity of three RAS axes. The effects of the increased concentration of glucose on insulin production/secretion were assessed in parallel to glucose-dependent alterations in the expression and activity of local pancreatic islet RAS and β-cell function. The findings of the present study suggest a shift from the classical ACE/Ang II/AT1R axis to the Ang- ( 1 - 7 ) - and Ang IV-triggered alternative RAS pathways.

## Materials and methods

### Cultivation and treatment of BRIN-BD11 cells

The BRIN-BD11 cells were cultured in Dulbecco’s modified Eagle’s medium containing 5.5 mM glucose, 4 mM L-glutamine, 10% (v/v) fetal calf serum, 100 U/ml penicillin and 100 μg/ml streptomycin at 37°C, 5% CO _2_ in a humidified atmosphere for 18–24 h prior to the experiments (all reagents from PAA Laboratories, Pasching, Austria). The cells were exposed to various concentrations of glucose (5.5, 11, 15 and 25 mM) or to 40 mM potassium chloride (KCl) in combination with 15 mM glucose and were incubated under the conditions described above for the periods of time indicated in the figure legends. For insulin secretion and expression analyses, in each case 2 million cells in 6 ml medium were seeded into 60×15 mm cell culture petri dishes; for the determination of enzyme activities, 0.5 million cells in 4 ml medium were seeded per well of a 6-well plate.

### RNA preparation and reverse transcription quantitative PCR (RT-qPCR)

RNA was extracted using the innuPrep RNA Mini kit (Analytik Jena, Jena, Germany) following the manufacturer’s instructions and the concentration was measured with a spectrophotometer NanoDrop 2000c (Thermo Fisher Scientific, Wilmington, DE, USA). RNA (1 μg) was reverse-transcribed using a Revert Aid™ First Strand cDNA Synthesis kit (Thermo Fisher Scientific, Braunschweig, Germany) following the instruction manual using oligo(dT) primers in a 30 μl reaction mixture. RT-qPCR was performed in a CFX96 thermocycler (Bio-Rad, Munich, Germany). A typical 20 μl reaction mixture consisted of 1X SensiMix™ SYBR Hi-ROX Mastermix (Bioline, Luckenwalde, Germany), 250–1,000 nM primer mix (sense and antisense) and 1 μl cDNA. Initial denaturation at 95°C for 10 min was followed by 45 cycles at 95°C for 10 sec, 57–65°C for 15 sec and 72°C for 30 sec. Melt curve analysis of the amplificates was carried out at 65–95°C with ΔT = 0.5°C every 5 sec. Data obtained by RT-qPCR were evaluated using the ΔΔ Cq-method included in the CFX96™/C1000 RT-qPCR detection system evaluation-software (Bio-Rad). Ribosomal protein L13a (Rpl13a) was used for normalization. The size and the purity of the PCR products were determined by melt curve analysis and by visualization on RedSafe™-stained agarose gels (iNtRON Biotechnology, Seoul, Korea). Amplificates having a Cq value >39 were considered as not expressed. Primers were designed using the Invitrogen OligoPerfect Designer and were obtained from Invitrogen (Darmstadt, Germany). Primer sequences (sense and antisense), the size of the amplificates in base pairs (bp) and the optimised annealing temperatures were as follows: ACE, 5′-AGTGGGTGCTGCTCTTCCTA-3′ and 5′-ATGGG ACACTCCTCTGTTGG-3′, 188 bp, 57–65°C; ACE2, 5′-GTGGAGCACTGACTGGAGC-3′ and 5′-GACAGGA GGCTCGTAAGGTG-3′, 403 bp, 59°C; APA, 5′-CCTCAC ATCCGGTGGTTGTC-3′ and 5′-TGGGTGACGTT CTGCTTTCC-3, 304 bp, 61°C; APN, 5′-CATCATAGCTCT GTCGGTGG-3′ and 5′-AGCGGACAGTACTGGAACC-3′, 238 bp, 61°C; AT1aR, 5′-CAGCGTGAGCTTCAACCTC TAC-3′ and 5′-CAGCCAGATGATGATGCAGGTG-3′, 145 bp, 61°C; AT1bR, 5′-TGTTGACAAGCCTGCGTGTGAC-3′ and 5′-GACATTGTGGACACCGCTATGC-3′, 165 bp, 61°C; AT2R, 5′-CACACTACGGAGCTTCTGTTGG-3′ and 5′-TTGGATGCTCTGACCTGGATGG-3′, 165 bp, 61°C; insulin 1, 5′-GCCCAGGCTTTTGTCAAACAG-3′ and 5′-GCAGATGCTGGTGCAGCACTG-3′, 237 bp, 57°C; insulin 2, 5′-CAGCACCTTTGTGGTTCTCAC-3′ and 5′-CAGTGCCAAGGTCTGAAGGTC-3′, 165 bp, 57°C; IRAP, 5′-GCCTACATCCAAACCTAACCTC-3′ and 5′-GCAG ATCTTGCTGCCAAAGG-3′, 367 bp, 57–65°C; Mas, 5′-CAGATGTCACCGCCCCAAGCA-3′ and 5′-GTGTTGCC ATTGCCCTCCTGA-3′, 534 bp, 62°C; NEP, 5′-CCAGACT GATTCGTCAGGAAC-3′ and 5′-CGGCTGAGGCTGC TTACAAG-3′, 397 bp, 57–65°C; Rpl13a, 5′-CTGGTACTTCC ACCCGACCTC-3′ and 5′-GGATCCCTCCACCCTAT GACA-3′, 131 bp, 57–65°C.

### Protein preparation and analyses

The cells were washed with ice-cold PBS and collected by centrifugation (1,900 × g, 4°C, 5 min). Cells were homogenised in lysis buffer, which contained 50 mM Tris-HCl (pH 7.5), 100 mM NaCl, 5 mM EDTA, 0.5% Triton X-100, 10% glycerol, 10 mM K _2_ HPO _4_ , 0.5% NP-40, 1 mM PMSF, 1 mM sodium vanadate, 0.5% desoxycholate, 20 mM NaF, 20 mM glycerol-2-phosphate (all from Sigma, Heidelberg, Germany) and a protease inhibitor cocktail (Roche, Mannheim, Germany), kept on ice for 30 min, frozen in liquid nitrogen, defrosted on ice and centrifuged at 16,000 × g and 4°C for 30 min to separate the proteins from the cell debris. Protein concentrations were determined using the Bradford method ( 41 ) . A total of 20 μg (40 μg for Mas-detection) of protein in a final volume of 30 μl 1X Laemmli buffer were separated by SDS-PAGE and transferred onto nitrocellulose (NC; Whatman, Dassel, Germany) or polyvinylidenefluoride (PVDF) membranes (Mas; Millipore, Bedford, MA, USA). The membranes were incubated with primary antibodies followed by incubation with horse radish peroxidase-conjugated secondary antibodies. For detection, the SuperSignal ^®^ West Dura Enhanced Chemiluminescence Substrate (Pierce, Rockford, IL, USA) was used. Subsequently, protein amounts were normalised to ribosomal protein, large, P0 (RPLP0) or actin signals. Protein expression was quantified using ImageJ software (Wayne Rasband, National Institute of Mental Health, Bethesda, MD, USA). The antibodies used, antibody dilutions and dilution buffers for western blotting were as follows: rabbit anti ACE2 (LS-B439; Biozol, Eching, Germany) 1:10,000, mouse anti-NEP (CD10; ab951) 1:1,000, goat anti-APA (BP1; ab36122) 1:2,000, rabbit anti-APN (CD13; ab108310; all from Abcam Cambridge, UK) 1:2,000, rabbit anti-IRAP (#6918; NEB; Cell Signaling Technology, Frankfurt/Main, Germany) 1:2,000, all in TBST/5% BSA/0.03% NaN _3_ ; rabbit anti-Mas (LS-B2564; Biozol) 1:2,000 in PBS/5% skimmed milk/0.03% NaN _3_ ; goat anti -actin (I-19, SC-1616; Santa Cruz Biotechnology, Inc., Heidelberg, Germany) 1:500, rabbit anti-RPLP0 (Biozol) 1:2,000, both in TBST/5% BSA/0.03% NaN _3_ . HRP-coupled secondary antibodies (all purchased from Cell Signaling Technology) were used in the following dilutions in 1X Roti ^®^ -Block (Carl Roth, Karlsruhe, Germany): anti rabbit IgG 1:10,000 (1:5,000 for Mas-detection), anti-mouse and anti-goat IgG 1:10,000.

### Determination of insulin secretion

Insulin concentrations in the supernatants of BRIN-BD11 cells were determined by a sandwich enzyme-linked immunosorbent assay (ELISA; Mercodia, Uppsala, Sweden) following the recommendations of the manufacturer.

### Enzymatic activities

The enzymatic activity of ACE, ACE2, APA, APN and IRAP was determined using viable BRIN-BD11 cells. The cells were scraped in PBS, centrifuged at 2,500 × g and resuspended in PBS. The following substrates were used for the determination of enzymatic activity: H-Ala-pNA-HCl for APN; H-Glu-pNA-2HCl (both from Bachem, Weil am Rhein, Germany) for APA; H-Leu-pNA-HCl (Sigma, Taufkirchen, Germany) plus RB3014 [specific inhibitor of APN, kindly provided by B.P. Roques (Département de Pharmacochimie Moléculaire et Structurale INSERM U266 - CNRS UMR 8600 UFR des Sciences Pharmaceutiques et Biologiques, Paris, France) ( 42 ) ] for IRAP; Mca-APK (Dnp; Enzo/Biomol, Hamburg, Germany) for ACE2. All the assays were carried out in 50 mM HEPES buffer containing 200 mM NaCl, 10 μM ZnCl _2_ and 1% DMSO pH 6.8. The enzyme reactions were performed in 1.5 ml tubes or in flat-bottom 96-well microtiter plates (Greiner Bio-One, Frickenhausen, Germany) for the measurement of the supernatants. Black plates were used for the measurement of fluorescence (ACE2) and transparent plates for the measurement of optical density. The total reaction volume amounting to 200 μl consisted of 100 μl buffer, 50 μl cell suspension and 50 μl substrate. The reaction was started by the addition of the substrate. For the determination of IRAP activity, a pre-incubation with the inhibitor, RB3014, was performed for 30 min at 37°C prior to the addition of the substrate. The optical density or fluorescence measured without cells was used as the blank sample. Following incubation at 37°C for 1 h in the dark and centrifugation at 2,500 × g for 5 min, the optical density of the supernatants was measured at 405 nm with 620 nm reference wavelength by a microtiter plate reader Infinite M200 (Tecan, Crailsheim, Germany). The fluorescence intensity in the ACE2 assay was determined using an excitation wavelength of 320 nm and an emission wavelength of 405 nm using a microtiter plate reader (Infinite F200; Tecan). All samples were determined in duplicates.

### Statistical analysis

Statistical analyses were carried out using GraphPad Prism 5 software (GraphPad, La Jolla, CA, USA). Data with n≥12 were analysed using one-way ANOVA to compare the means of 3 or more unmatched groups. If there was a statistically significant difference, unpaired t-tests were applied to compare 2 sets of measurements. Normally distributed data are illustrated as the means + SEM.

In order to compare 3 or more groups of data (n≥4 to <12), the non-parametric Kruskal-Wallis test was used. If there was a significant difference, Mann-Whitney tests were applied to compare 2 sets of measurements. Non-parametric data are illustrated as boxplots with medians, quartiles and an interquartile range (IQR) ± 1.5 × IQR. P-values <0.05 were considered to indicate statistically significant differences.

## Results

### Effect of glucose on insulin production and secretion

**Figure 2:**
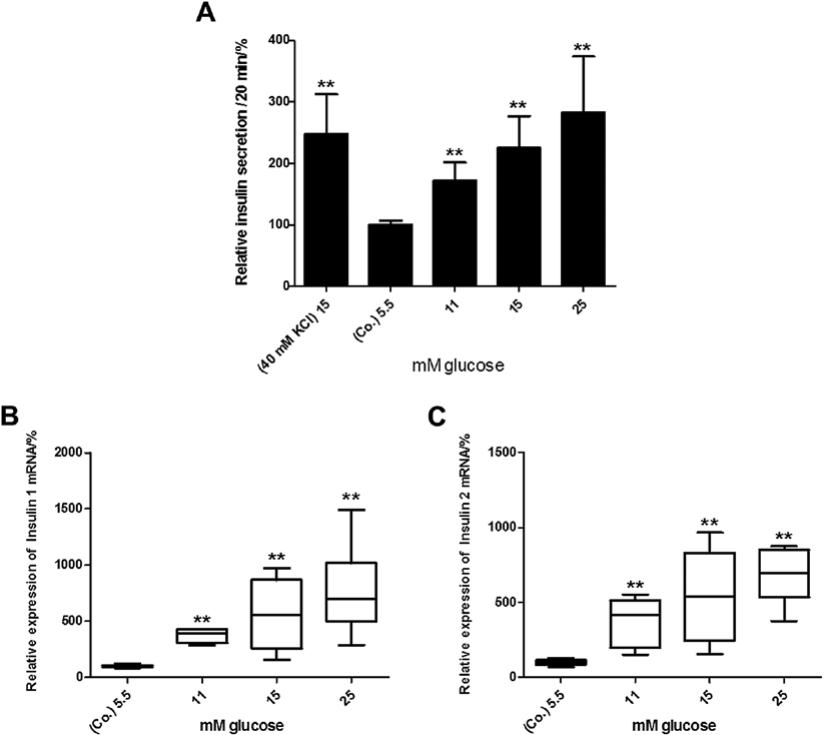
Glucose increases (A) insulin secretion and the mRNA levels of (B) insulin 1 and (C) insulin 2 in a dose-dependent manner in BRIN-BD11 cells. (A) Insulin levels were quantified in the supernatants by ELISA 20 min after stimulation. Data are presented as the means + SEM of 13 independent experiments. 100% = 0.13 μg/l insulin [t-test ^**^ P<0.01 vs. control (Co.)]. (B) mRNA levels were quantified by quantitative PCR after 24 h of incubation. Data were evaluated and normalized to Rpl13a mRNA expression using the ΔΔ Cq method. Data are presented as box plots with medians, quartiles and an interquartile range (IQR) ± 1.5 × IQR of 5 independent experiments [Mann-Whitney ^**^ P<0.01 vs. control (Co.)]. ELISA, enzyme-linked immunosorbent assay.

To verify that BRIN-BD11 cells can be used as a suitable β-cell model, the short-time glucose-stimulated insulin secretion (GSIS) was determined. Basal levels of insulin in the cell culture supernatants (0.13 μg/l) were set to 100% (control). Stimulation with 40 mM KCl and 15 mM glucose serving as the positive control increased the amounts of insulin to ~247±65% (P<0.01) of the control ( Fig. 2A ). In response to a 20-min exposure of the BRIN-BD11 cells to elevated glucose concentrations, there was a dose-dependent increase in insulin secretion ( Fig. 2A ). This increase was significant from the concentration of 11 mM glucose (172±31%, P<0.01) and reached 283±91% (P<0.01) with 25 mM glucose ( Fig. 2A ). Furthermore, the BRIN-BD11 cells, when exposed to elevated glucose concentrations for 24 h, showed a dose-dependent increase in insulin 1- and 2 mRNA levels ( Fig. 2B and C ). Again, this effect was significant from the concentration of 11 mM glucose, reaching 389% (Q1 = 352; Q3 = 412) (P<0.01) for insulin 1 mRNA or 415% (203; 510) (P<0.01) for insulin 2 mRNA ( Fig. 2B and C ).

### Basal RAS expression

**Figure 3:**
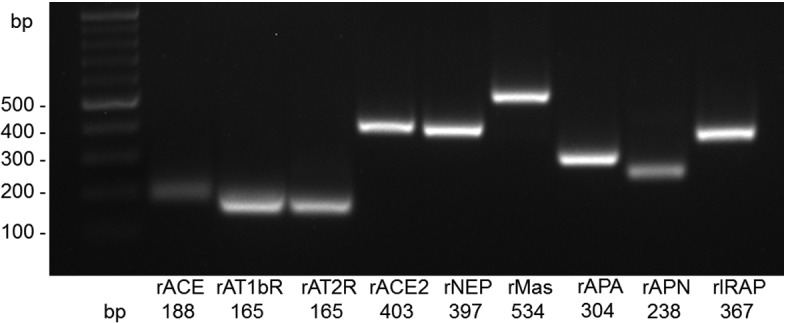
BRIN-BD11 cells express essential components of the renin-angiotensin system. PCR products were visualized by agarose gel electrophoresis and RedSafe™ staining. The size of amplificates is given in base pairs (bp). AT1bR, angiotensin II type 1b receptor; AT2R, angiotensin II type 2 receptor; ACE, angiotensin-converting enzyme; NEP, neutral endopeptidase 24.11; Mas, Mas receptor; APA/APN, aminopeptidases A and N; IRAP, insulin-regulated aminopeptidase.

After having confirmed that BRIN-BD11 rat insulinoma cells exhibit an appropriate responsiveness to glucose, we then determined the mRNA expression levels of components of the main RAS axes. BRIN-BD11 cells express all essential components of RAS investigated, including the constituents of the classical RAS axis, ACE, AT1bR, AT2R (but not AT1aR), as well as those of the alternative APN/Ang IV/IRAP and ACE2/Ang- ( 1 - 7 ) /Mas axes ( Fig. 3 ).

### Effect of glucose on RAS expression and activity

**Figure 4:**
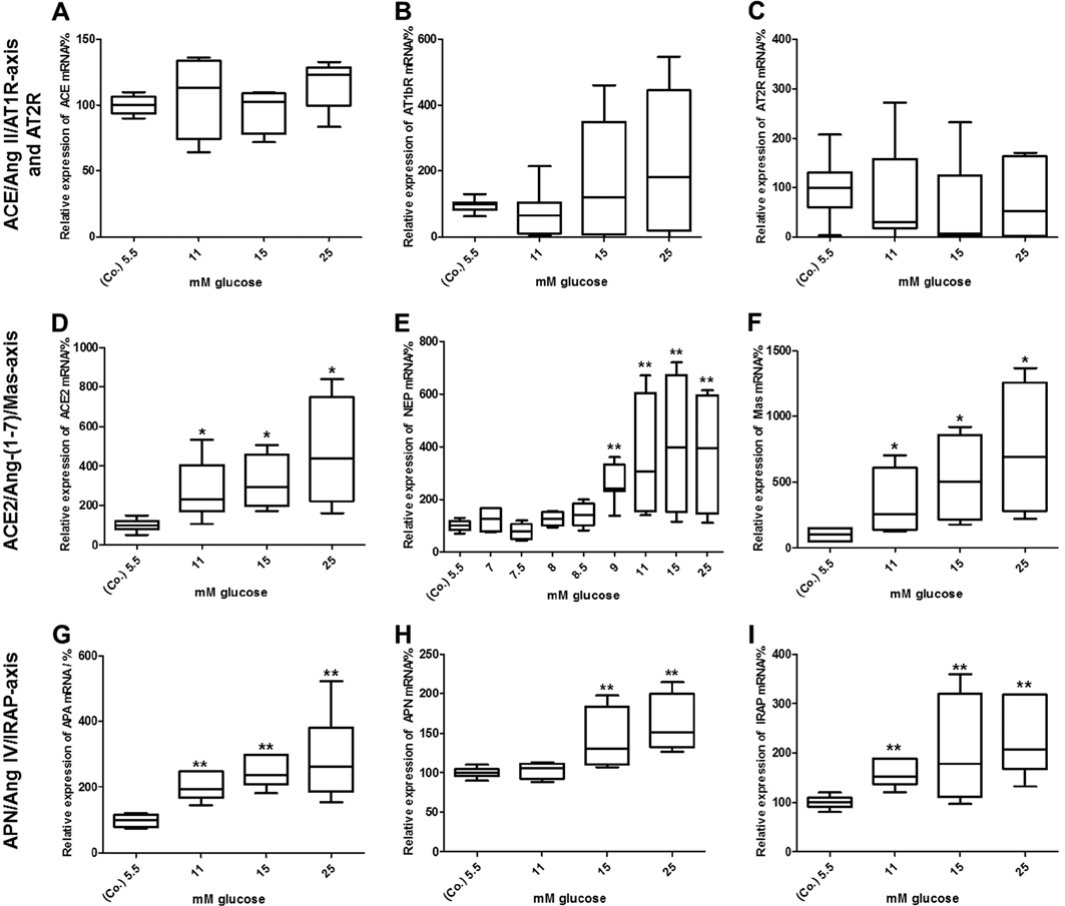
Effects of glucose on the mRNA expression of renin-angiotensin system (RAS) components. Elevated concentrations of glucose did not alter the mRNA levels of (A) angiotensin-converting enzyme (ACE), (B) angiotensin II type 1b receptor (AT1bR), or (C) angiotensin II type 2 receptor (AT2R) mRNA levels in BRIN-BD11 cells after 24 h, whereas the levels of (D) ACE2, (E) neutral endopeptidase 24.11 (NEP), (F) Mas, (G) aminopeptidase A (APA), (H) aminopeptidase N (APN) and (I) insulin-regulated aminopeptidase (IRAP) were dose-dependently increased. mRNA levels were determined by quantitative PCR. Data were evaluated and normalized to Rpl13a mRNA expression using the ΔΔ Cq method. Data are presented as box plots with medians, quartiles and an interquartile range (IQR) ± 1.5 × IQR of 5 independent experiments [Mann-Whitney ^*^ P<0.05, ^**^ P<0.01, vs. control (Co.)].

We then determined whether the exposure of BRIN-BD11 cells to increasing concentrations of glucose alters the expression and, if applicable, the enzymatic activity of RAS components. As shown in Fig. 4A–C , the mRNA expression of constituents of the classical RAS axis, ACE and AT1bR, as well as those of AT2R was not altered after 24 h. By contrast, glucose at higher concentrations induced a dose-dependent increase in the mRNA expression of every component analysed within the ACE2/Ang- ( 1 - 7 ) /Mas axis ( Fig. 4D–F ). More precisely, with the concentration of 11 mM glucose a significant increase in ACE2 [245% (202; 410) , P<0.05], NEP [305% (188; 604) , P<0.01] and Mas [255% (164; 665) , P<0.05] mRNA expression was observed. The mRNA levels even further increased in response to higher concentrations of glucose. In the case of NEP, glucose was additionally titrated between 5.5 and 11 mM, as there was a marked increase in mRNA expression between these 2 concentrations ( Fig. 4E ). As observed for the ACE2/Ang- ( 1 - 7 ) /Mas axis, glucose also increased the mRNA expression of APA, APN and IRAP, which are all constituents of the APN/Ang IV/IRAP alternative RAS pathway ( Fig. 4G–I ). Whereas the mRNA levels of APA [194% (177; 248) , P<0.01] and IRAP [151% (143; 185) , P<0.01] were already elevated in response to 11 mM or higher concentrations of glucose, APN mRNA levels [130% (116; 179) , P<0.01] were significantly upregulated from starting from the concentration of 15 mM glucose.

**Figure 5:**
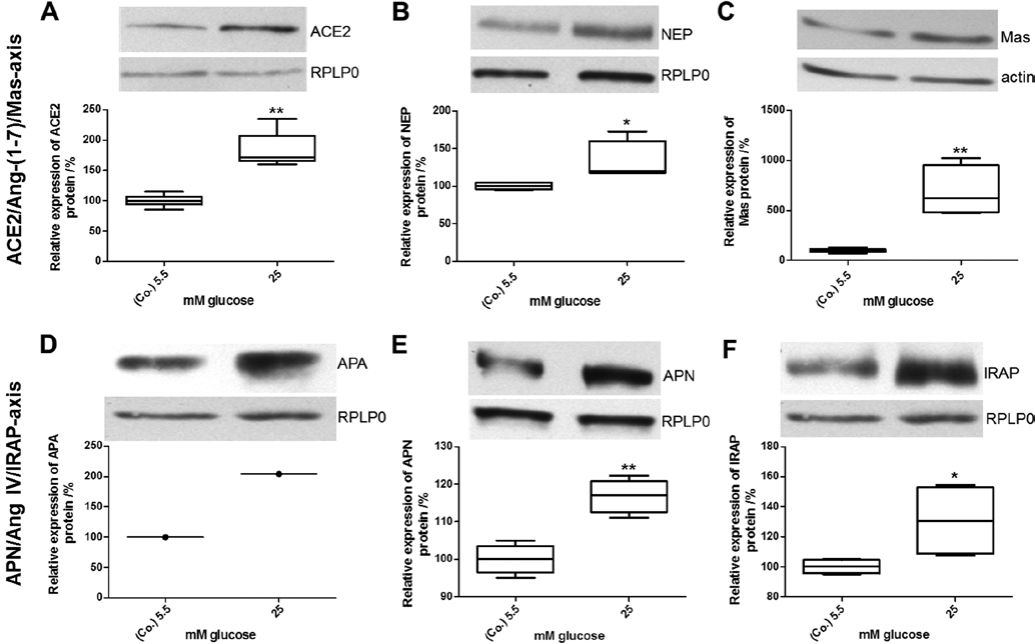
Effects of glucose on protein expression of renin-angiotensin system (RAS) components. Glucose increased the levels of (A) angiotensin-converting enzyme (ACE)2, (B) neutral endopeptidase 24.11 (NEP), (C) Mas receptor, (D) aminopeptidase A (APA), (E) aminopeptidase N (APN) and (F) insulin-regulated aminopeptidase (IRAP) protein in BRIN-BD11 after 24 h. Twenty micrograms of total protein (40 μg for Mas) were separated by SDS-PAGE and visualized by western blot analysis. Expression data were quantified densitometrically and normalized to actin or RPLP0. Shown is 1 representative western blot out of 4 and the related box plots with medians, quartiles and an interquartile range (IQR) ± 1.5 × IQR, respectively [Mann-Whitney ^**^ P<0.01, ^*^ P<0.05, vs. control (Co.)].

Changes in the expression of constituents of the alternative RAS axes observed at the mRNA level were subsequently verified at the protein and enzymatic activity level. Immunoblot analyses revealed a dose-dependent increase in the levels of ACE2, NEP, Mas, APA, APN and IRAP protein in response to the exposure of the BRIN-BD11 cells to 25 mM glucose for 24 h. As shown in Fig. 5 , the levels of ACE2, NEP and Mas increased to 172% [ (166; 206) , P<0.01], 120% [ (118; 160) , P<0.05] or 623% [ (482; 950) , P<0.01] compared with the control ( Fig. 5A–C ). Similarly, the protein levels of APA (204%), APN [109% (100; 143) , P<0.01] and IRAP [130% (109; 153) , P<0.05] were markedly increased by 25 mM glucose ( Fig. 5D–F ).

**Figure 6:**
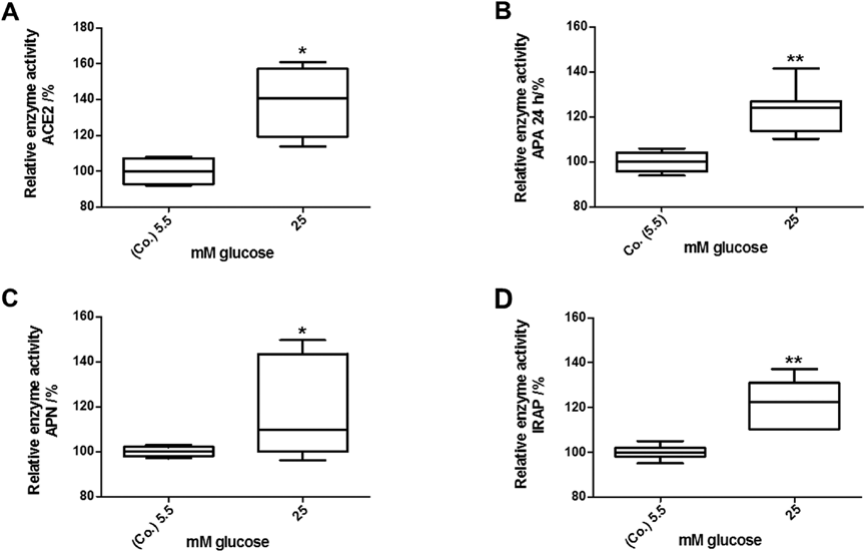
Effects of glucose on enzymatic activities of renin-angiotensin system (RAS) proteases. Exposure of BRIN-BD11 cells to elevated concentrations of glucose led to increased enzymatic activities of (A) angiotensin-converting enzyme (ACE)2, (B) aminopeptidase A (APA), (C) aminopeptidase N (APN) and (D) insulin-regulated aminopeptidase (IRAP) after 24 h. Protease activities were determined by the incubation of viable cells with chromogenic or fluorogenic (ACE2) substrates. The activities were calculated per one million cells. Illustrated are boxplots with medians, quartiles and an interquartile range (IQR) ± 1.5 × IQR of 4 independent experiments [Mann-Whitney ^**^ P<0.01, ^*^ P<0.05, vs. control (Co.)].

The results obtained by the analysis of mRNA and protein expression clearly suggest an induction of the two alternative RAS axes. To further support this view, the enzymatic activity of relevant RAS proteases under conditions of low and high glucose was measured. In full accordance with the observed changes in mRNA and protein expression levels, there was a significant increase in the enzymatic activities assigned to both the APN/Ang IV/IRAP axis (APA, APN and IRAP) and the ACE2/Ang- ( 1 - 7 ) /Mas axis (ACE2). More precisely, the enzymatic activities increased with the concentration of 25 mM glucose as follows: ACE2, 141% [ (119; 157) , P<0.05]; APA, 124% [ (114; 127) , P<0.01]; APN, 109% [ (100; 143) , P<0.05]; and IRAP, 123% [ (111; 131) , P<0.01] ( Fig. 6 ).

## Discussion

Glucose and fatty acids represent the main nutritional components. Their concentration in the blood differs between food intake and fasting periods to a large extent. Normally, the acute exposure of β-cells to elevated concentrations of glucose following food intake leads to an adequate insulin secretion. However, a sustained elevation of plasma levels of glucose as in the case of patients suffering from impaired glucose tolerance or DM may adversely affect islet function. Accumulating evidence indicates that a systemic and/or local activation of classical RAS is common to various diabetic risk factors, including hypertension and obesity ( 43 , 44 ) . Ang II, in particular, has been reported to impair insulin signaling and islet function ( 45 , 46 ) . Although first data suggest a protective role of alternative RAS axes on β-cell function, this needs to be further substantiated. Therefore, in this study, we aimed i) to assess the basal expression levels and enzymatic activity of the components of three major RAS axes in BRIN-BD11 insulinoma cells and ii) to evaluate possible alterations thereof in response to the exposure of the cells to high glucose concentrations. A crucial limitation when using immortalised β-cell lines as a model system may arise from their lack of an adequate responsiveness to glucose. The data from the present study demonstrate an appropriate preservation of functionality in BRIN-BD11 cells, showing a dose-dependent short-term insulin secretion following exposure to various concentrations of glucose. These findings are in full accordance with previous results from the study by McClenaghan *et al* ( 47 ) , who established this cell line and confirmed that BRIN-BD11 cells are a suitable model for the study of β-cell function.

In this study, we first demonstrated (on different levels of expression) that the essential components of the three main RAS axes are expressed in BRIN-BD11 cells under basal conditions. In detail, we proved the following constituents of RAS to be present in the BRIN-BD11 rat insulinoma cell line: ACE, AT1bR, AT2R, ACE2, NEP, Mas, APA, APN and IRAP, but not AT1aR. These results are in agreement with those of previous studies, demonstrating the expression of (pro)renin ( 48 , 49 ) , angiotensinogen ( 50 – 52 ) , ACE ( 49 – 53 ) and ACE2 ( 49 ) , AT1R ( 48 , 50 , 53 ) and AT2R ( 53 , 54 ) , Mas ( 55 ) and IRAP ( 56 , 57 ) in several islet-specific cell types, specifically in β-cells, in various species including humans, rats, mice and canines ( 28 ) . In this study, to our knowledge, we demonstrate for the first time the expression of APA, APN and NEP in the BRIN-BD11 pancreatic β-cell line, thus proving the presence of local RAS on β-cells.

Despite the earlier detection of RAS components in pancreatic islets, Lupi *et al* ( 58 ) were the first to study the effects of glucose on RAS mRNA expression in islets of Langerhans. A second study confirmed these findings, showing that in INS-1E rat insulinoma cells, chronic hyperglycaemia induces AT1R gene and protein expression, in parallel to enhanced ROS production and impaired GSIS ( 59 ) . As regards other cell types, hyperglycaemia has been shown to increase extracellular matrix (ECM) production through RAS activation in rat pancreatic stellate cells (PSCs), which are involved in pancreatic inflammation and fibrosis ( 60 ) . In these cells, 27.7 mM glucose induced an increase in ACE and AT1aR mRNA expression after 72 h, whereas AT1bR and AT2R mRNA levels remained unaltered ( 61 ) . Again, ACE _i_ s and ARBs prevented the increase in proliferation and ECM production, emphasizing the pivotal role that Ang II plays in the pathophysiology of pancreatic inflammation and fibrosis, which are exacerbated by chronic hyperglycaemia ( 60 ) . The increased mRNA expression and enzymatic activity of NEP have been observed in human microvascular endothelial cells (HMECs) in response to 40 mM glucose. ROS seems to play a role in this process, since vitamins C and E attenuate the effects of glucose ( 61 , 62 ) . In rats fed a high sucrose diet for 30 days, Ang- ( 1 - 7 ) -levels, ACE2, AT1R and AT2R protein expression have been shown to increase in adipose tissue, but remain unaltered in the kidneys. By contrast, ACE activity is decreased in adipose tissue, but increased in the kidneys ( 63 ) .

In this study, we examined the effects of glucose on the expression and/or enzymatic activities of the β-cell RAS components in BRIN-BD11 cells. To the best of our knowledge, this is the first comprehensive study of islet RAS i) considering both the classical and the alternative axes of RAS and ii) analyzing RAS at different expression and enzymatic activity levels in parallel. Of note, under conditions of high glucose expression and activities of the alternative RAS axes, ACE2/Ang- ( 1 - 7 ) /Mas and APN/IRAP levels were found to increase after 24 h. In contrast to this, the classical ACE/Ang II/AT1R axis and AT2R remained largely unaffected by glucose. Of note, in isolated human islets, ACE and AT1R mRNA levels have been shown to be upregulated by 22.2 mM glucose ( 58 ) . The extent to which islet-specific cell types other than β-cells contribute to the ACE/Ang II/AT1R induction observed in whole islets remains to be elucidated. The data presented in this study suggest that in β-cells, there is a general shift from the classical to the alternative and supposedly protective RAS axes under acute hyperglycaemic conditions. Whereas 22.2 mM glucose have been shown to decrease insulin secretion by isolated human islets, possibly due to oxidative stress that was concomitantly observed ( 58 ) , in the present study, glucose at concentrations of 25 mM enhanced insulin expression and secretion in BRIN-BD11 cells. It can be speculated that in human islets, the increased expression and enzymatic activity of the classical Ang II/ACE/AT1R axis contributes through increased ROS production to the compromised insulin response under very high glucose conditions. This view is supported by the well-established fact that Ang II upon binding to the AT1R, induces the activation of NADPH oxidase and, thus, enhances the production of ROS. Recent evidence indicates that this mechanism works in islets or β-cells as well ( 64 – 66 ) . Accordingly, as shown in a previous study, impaired insulin secretion and elevated ROS production can be abolished by ACE _i_ s ( 58 ) . The authors concluded that ACE _i_ s protect human islets from apparent glucotoxicity ( 58 ) . It is tempting to speculate that BRIN-BD11 cells tolerate higher glucose concentrations well, as they show no such induction of the Ang II/ACE/AT1R axis. This hypothesis, however, needs to be verified in future studies. Furthermore, this may be true for the short-term exposure of β-cells to high glucose only, whereas sustained hyperglycaemia provokes opposite effects. In support of this view, chronically elevated glucose levels have been shown to increase Ang II levels in several models, including diabetic rats, thereby promoting Ang II/AT1R-mediated glucotoxicity and hampering islet blood flow, insulin production and GSIS ( 58 , 60 , 67 , 68 ) .

By contrast, the short-term exposure of BRIN-BD11 cells to high 25 mM glucose as performed in the present study did not compromise cell viability (data not shown) or insulin production/secretion. Our data clearly demonstrate a profound induction of the alternative ACE2/Ang- ( 1 - 7 ) /Mas and APN/Ang IV/IRAP axes in the BRIN-BD11 cells. The observed parallel increase in mRNA and protein expression together with corresponding enzymatic activities emphasise the relevance of this finding and point to a functional role of these alternative RAS axes in maintaining β-cell viability and function. As to how the activation of alternative RAS axes and, in particular, the observed Ang- ( 1 - 7 ) -mediated activation of PI3K via Mas aims to compensate Ang II/AT1R-mediated toxicity, remains to be fully elucidated ( 69 ) .
